# The Potential Role of Neurofilament Light in Mild Traumatic Brain Injury Diagnosis: A Systematic Review

**DOI:** 10.7759/cureus.31301

**Published:** 2022-11-09

**Authors:** Cullen D Farragher, Ying Ku, James E Powers

**Affiliations:** 1 Emergency Medicine, Campbell University School of Osteopathic Medicine, Lillington, USA; 2 Plastic and Reconstructive Surgery, Campbell University School of Osteopathic Medicine, Lillington, USA; 3 Emergency Medicine/Clinical Integration, Campbell University School of Osteopathic Medicine, Lillington, USA

**Keywords:** mild traumatic brain injury, traumatic brain injury, sport-related concussion, concussion diagnosis, neurofilament light (nf-l), serum biomarkers, brain concussion, mild traumatic brain injury (mtbi)

## Abstract

Mild traumatic brain injury (mTBI) is an insult to the CNS often overlooked at the time of presentation due to variable symptomatology and undetectable nature on CT/MRI. Increased exposure to repetitive head injuries results in a high prevalence of mTBI among athletes and military personnel. While most patients fully recover with rest, some are at risk for long-lasting neurocognitive dysfunction, leading to a high morbidity and cost burden on the healthcare system. Currently, there are no unified symptom-based criteria or gold standard objective measurement for mTBI. Neurofilament light (Nf-L) is a highly sensitive biomarker for axonal injury with the potential to serve as an objective serum measurement for mTBI. This systematic review investigates the ability of Nf-L to accurately diagnose acute mTBI in athletes and military personnel.

A comprehensive literature search of PubMed, Scopus, and Google Scholar from 2010 to 2021 using keywords neurofilament light chain, mTBI, concussion, athletes, and military identified 239 articles for eligibility screening. Ten articles met the inclusion criteria for qualitative analysis, with extracted data including Nf-L levels, recovery characteristics, and neuroimaging results. Of the 10 studies meeting inclusion criteria, one was military-related, five were sports-related, and four were mixed-focus. Six studies investigated the association between mTBI and Nf-L levels within 24 hours of injury. Four of these studies involved athletes, with three showing evidence of acute Nf-L elevations. No evidence of acute Nf-L elevations was reported among military personnel or emergency department patients. Nf-L elevations were recorded at various time points greater than 24 hours post-injury in athletes (two studies) and emergency department patients (one study). Positive associations were found between Nf-L levels and loss of consciousness/post-traumatic amnesia (one study), positive neuroimaging findings (three studies), and prolonged recovery times (three studies).

We are unable to conclude whether Nf-L has the capacity for acute diagnosis of mTBI or the optimal time for serum measurement. Nf-L does, however, shows promise as a prognostic factor for mTBI complications, neuroimaging findings, and recovery. Additional studies are warranted, as the use of Nf-L in early diagnosis of mTBI in the future would improve clinical management while decreasing complications and healthcare costs.

## Introduction and background

Mild traumatic brain injury (mTBI), known colloquially as concussion, continues to be a major health concern in the United States due to the high incidence and expense burden on the healthcare system. Traumatic brain injuries account for approximately 2.5 million emergency department visits annually, with an average cost of $800 per concussion [1,2]. As many as 5.3 million Americans suffer prolonged disability as a result of such injuries [3]. mTBI is especially prevalent among contact-sport athletes and military service members due to increased exposure to repetitive head injuries. An estimated 3.8 million sports-related mTBIs occur annually in the US [4], while more than 430,000 military personnel were diagnosed with mTBI between 2000 and 2020 [5].

mTBI is a form of head trauma characterized by microstructural damage to brain tissue as a result of external shear stress [6,7]. Such microscopic changes are often undetectable on standard computed tomography (CT) and magnetic resonance imaging (MRI) [8]. As a result, physicians often rely solely on the subjective symptoms reported by patients. mTBI diagnosis is complicated by the non-specific nature of the most commonly reported symptoms, including headache, dizziness, confusion, irritability, impaired concentration, or insomnia [6,9,10]. Loss of consciousness or amnesia may also manifest in some cases [6], with as many as 15% of patients experiencing long-term neurocognitive dysfunction following diagnosis [11]. Several assessment tools exist to assist with mTBI diagnosis, including the Standardized Assessment of Concussion (SAC) test, Sport Concussion Assessment Tool 5 (SCAT5), and Military Acute Concussion Evaluation (MACE), among others [12]. Although these tools are convenient in the acute setting, such rapid screening does not account for the variation in symptomatology between patients. With no unified symptom-based criteria or gold standard objective measurement for mTBI currently available, mTBI is often overlooked and consequently underdiagnosed.

Biomarkers are objective physiological indicators of disease or injury that, in the case of mTBI, have the potential to enable accurate and definitive diagnosis in the acute clinical setting [13]. Biomarkers that have been previously studied in the context of mTBI include glial fibrillary acidic protein (GFAP), S100β, neuron-specific enolase (NSE), ubiquitin C-terminal hydrolase L1 (UCH-L1), tau, alpha-II spectrin, and neurofilament light (Nf-L) [14,15]. Nf-L is of particular interest as it is a major structural protein located exclusively in axonal membranes and has been shown to have high sensitivity for axonal injury [15,16]. The shear stress on central nervous system axons from external forces disrupts axonal membranes, resulting in the release of Nf-L into the blood [17]. Measuring Nf-L levels in the blood may provide an opportunity or an objective measurement for mTBI diagnosis.

The objective of this review is to evaluate current research to determine if Nf-L has a potential role in accurately diagnosing mTBI within 24 hours of injury. The focus was placed on contact-sport athletes and military personnel due to the high prevalence of mTBI among these populations. Early objective diagnosis of mTBI would improve clinical management and decrease mTBI complications, morbidity, and ultimately healthcare cost for both patients and medical facilities.

## Review

Methods

Search Strategy and Study Selection

A comprehensive search of PubMed, Scopus, and Google Scholar was conducted to identify studies that explored the use of Nf-L in diagnosing mTBI in an acute setting. The authors define the acute setting to be within 24 hours of injury. The search adhered to the 2020 Preferred Reporting Items for Systematic Reviews and Meta-Analyses (PRISMA) guidelines [18]. The initial search was performed between October 2020 and February 2021, with a final search in July 2021 for any additional, newly published studies. Literature was filtered such as to isolate those written in English and published within the past 11 years (2010 through July 2021). The following Medical Subject Heading (MeSH) terms and keywords were utilized to locate studies of interest: brain concussion [MeSH], neurofilament proteins [MeSH], neurofilament protein L [Supplementary Concept], neurofilament light chain, brain injury, mild traumatic brain injury (mTBI), concussion, athlete, and military [keywords].

**Figure 1:**
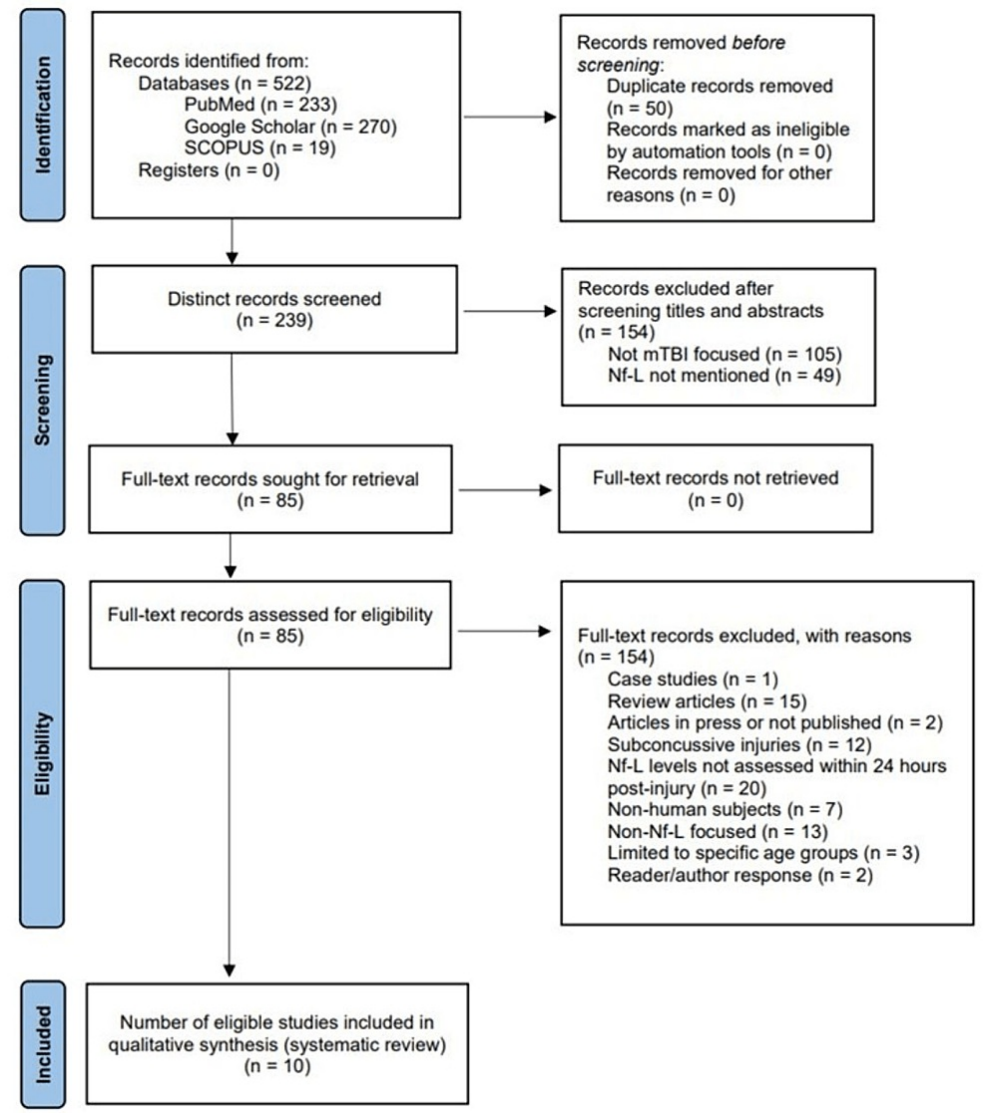
Article selection process Diagram depicting the selection process following the 2020 PRISMA guidelines for studies in this systematic review, including databases used and exclusion criteria [18]. Abbreviations: PRISMA, Preferred Reporting Items for Systematic Reviews and Meta-Analyses; n, number of studies; mTBI, mild traumatic brain injury; Nf-L, neurofilament light.

After removing duplicates, all titles and abstracts yielded by the search criteria were divided and screened independently by two authors (C. Farragher and Y. Ku) to ensure inclusion and exclusion criteria were met (Figure 1). Articles were removed if mTBI was not the main focus or if no data on Nf-L were provided. Full-text assessment of all remaining articles for eligibility was performed by both authors independently. Case studies, literature reviews, and unpublished manuscripts were excluded. Additionally, studies were excluded for the following reasons: Nf-L was not measured within 24 hours of injury, the focus was on subconcussive injuries or non-human subjects, or the sample was limited to a specific age group. A consensus was reached through discussion between the two authors in cases of disagreement regarding study eligibility. More detailed information regarding the study selection process can be found in Figure 1.

Quality Assessment

All final selected studies were evaluated to assess the risk of bias using the National Institutes of Health (NIH) Study Quality Assessment Tools [19]. This assessment includes the following domains: research question, study population, groups recruited from the same population and uniform eligibility criteria, sample size justification, exposure assessed prior to outcome measurement, sufficient timeframe to see an effect, different levels of the exposure of interest, exposure measures and assessment, repeated exposure assessment, outcome measures, blinding of outcome assessors, follow-up rate, and statistical analyses. Each study was assessed independently by the two authors. Discussion after the assessment was conducted to reach a consensus regarding the quality of the selected studies. Studies received up to one point for meeting the criteria within each of the questions established by the NIH Study Quality Assessment Tools [19], with a maximum score of 14 for cohort and cross-sectional studies and 12 for case-control studies. Studies were assigned quality scores of good (cohort/cross-sectional: 11-14; case-control: 10-12), fair (cohort/cross-sectional: 7-10; case-control: 7-9), or poor (cohort/cross-sectional/case-control: 0-6).

Data Extraction and Analysis

Following the review of each selected study, the following data were collected: authors and year of publication, study type, subject numbers and characteristics, mechanism/activity of injury, and outcomes of interest. Data were analyzed and presented in a table format. Studies of similar focus were compared to reach conclusions regarding the use of Nf-L in the context of mTBI.

Results

Literature Search

Following the 2020 PRISMA guidelines [18], the literature search yielded 233 articles through the PubMed database and a combined 289 articles through a Scopus and Google Scholar search. After removing duplicates and performing an initial screen for eligibility, 85 articles were isolated and reviewed in full text. After further review, 75 articles were excluded for reasons specified in Figure 1. The remaining 10 studies were deemed eligible for final inclusion.

Methodological Quality of Selected Articles

**Figure 2:**
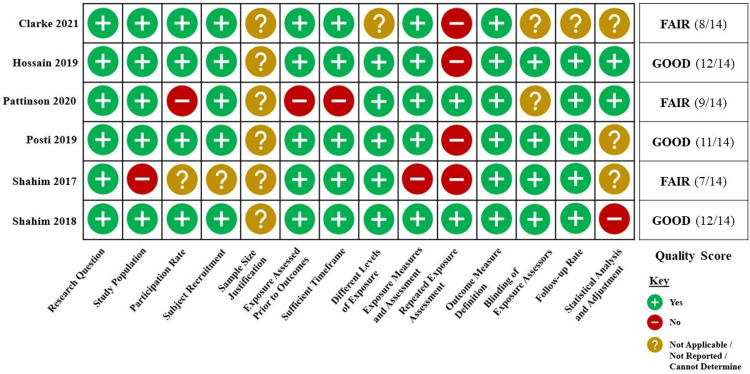
Quality assessment of cohort and cross-sectional selected studies Five cohort and one cross-sectional studies were included in this systematic review. Studies received up to one point for meeting the criteria within each of the 14 questions established by the National Institutes of Health (NIH) Study Quality Assessment Tools [19], with a maximum score of 14. Studies were given quality scores of good (11-14), fair (7-10), or poor (0-6). Citations: Clarke et al. [20], Hossain et al. [21], Pattinson et al. [22]. Posti et al. [23], Shahim et al. (2017) [24], and Shahim et al. (2018) [25].

**Figure 3:**
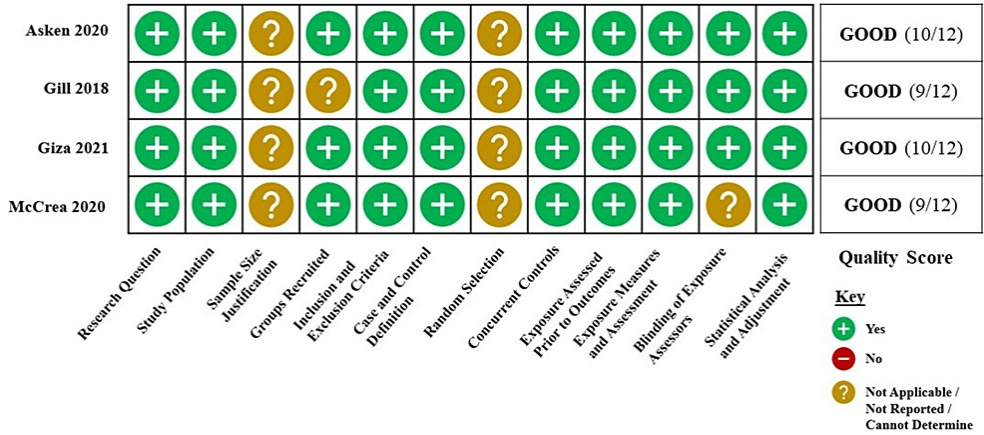
Quality assessment of case-control selected studies Four case-control studies were included in this systematic review. Studies received up to one point for meeting the criteria within each of the 12 questions established by the National Institutes of Health (NIH) Study Quality Assessment Tools [19], with a maximum score of 12. Studies were given quality scores of good (10-12), fair (7-9), or poor (0-6). Citations: Asken et al. [26], Gill et al. [27], Giza et al. [28], and McCrea et al. [29].

For the purposes of quality assessment, the 10 studies were divided into two groups, cohort and cross-sectional studies (Figure 2) and case-control studies (Figure 3), based on the study design categories established within the NIH Study Quality Assessment Tools [19]. All included studies were determined to be of good or fair quality.

Characteristics of Reviewed Studies

The selected articles included four case-control, one cross-sectional, and five cohort studies of varying focus in regard to patient populations and outcomes of interest. One was military-related, five were contact-sport-related, and four were of mixed-focus. The mixed-focus articles did not focus specifically on military personnel or athletes, however, provided valuable data regarding the relationship between Nf-L levels and CT and MRI results, injury complications, and patient recovery status. Information regarding the selected studies, study design, patient populations, and extracted data can be found in Table 1.

**Table 1:** Characteristics of reviewed studies A total of 10 studies were included in the qualitative synthesis of this systematic review. Abbreviations: CC, contact-control; CM, community-control; TC, trauma-control; NC, non-contact control; NA, non-athletic control; GC, gymnast control; mTBI, mild traumatic brain injury; Nf-L, neurofilament light; NIH, National Institutes of Health; MRI, magnetic resonance imaging; CT, computed tomography; RTP, return to play; LOC, loss of consciousness; PTA, post-traumatic amnesia.

Authors, year of publication	Study type	Number of subjects (n)	Patient demographics	Patient population	Mechanism of injury	Outcomes of interest
Asken et al. (2020) [26]	Case-control	n = 110 (28 concussed, 82 contact-control)	Median age (years): 19 (concussed), 20 (CC); male (%): 50 (concussed), 53.7 (CC)	Varsity athletes: University of Florida	Football, basketball, tennis, soccer, lacrosse, swim & dive	Nf-L levels (median 4 hours post-injury)
Clarke et al. (2021) [20]	Prospective cohort	n = 226 (76 concussed, 84 community-control, 52 trauma-control)	Mean age (years): 33.2 (concussed), 33.2 (CM), 32.4 (TC); male (%): 64.5% (concussed), 57.1 (CM), 51.9 (TC)	Patients at St. Olavs Hospital (Trondheim University Hospital) and Trondheim Municipal Emergency Clinic (Norway)	Not specified	Nf-L levels (<24 hours, 72 hours, 2 weeks, 3 months, 12 months post-injury); MRI results
Gill et al. (2018) [27]	Case-control	n = 323 (274 concussed, 49 control)	Mean age (years): 46.52 (concussed), 48.52 (control); male (%): 61 (concussed), 60.58 (control)	Concussed patients from participating NIH and non-NIH hospitals; healthy controls from the NIH database	Not specified	Nf-L levels (<48 hours post-injury); CT and MRI results
Giza et al. (2021) [28]	Case-control	n = 103 (67 concussed, 36 contact-control)	Mean age (years): 18.6 (concussed), 19.5 (CC); male (%): 59.7 (concussed), 69.4 (CC)	Military cadets: U.S. Military Academy at West Point, U.S. Air Force Academy	Combative training	Nf-L levels (<6 hours post-injury, 24-48 hours post-injury, asymptomatic, 7 days post-RTP)
Hossain et al. (2019) [21]	Prospective cohort	n = 105	Mean age (years): 47.64; male (%): 68.2	Patients at Turku University Hospital (Finland)	Not specified	Nf-L levels (<24 hours post-injury); recovery status: (complete/incomplete, favorable/unfavorable)
McCrea et al. (2020) [29]	Case-control	n = 504 (264 concussed, 138 contact-control, 102 non-contact control)	Mean age (years): 19.08 (concussed), 19.03 (CC), 19.39 (NC); male (%): 79.9 (concussed), 77.5 (CC), 80.4 (NC)	Contact and non-contact sport athletes: U.S. Military Academy at West Point, U.S. Air Force Academy, University of California Los Angeles, University of North Carolina, University of Wisconsin, Virginia Tech	Football, hockey, lacrosse, rugby, soccer, wrestling	Nf-L levels (<24 hours post-injury, 24-48 hours post-injury, asymptomatic, 7 days post-RTP); complications of initial injury (LOC/PTA)
Pattinson et al. (2020) [22]	Cross-sectional	n = 127	Mean age (years): 18.9; male (%): 76.4	Contact-sport athletes: U.S. Military Academy at West Point, U.S. Air Force Academy, University of California Los Angeles, University of North Carolina, University of Wisconsin, Virginia Tech	Football, soccer, lacrosse, hockey, rugby	Nf-L levels (<21 hours post-injury, 24-48 hours post-injury, asymptomatic, 7 days post-RTP); length of recovery (> or < 14 days)
Posti et al. (2019) [23]	Prospective cohort	n = 93 (55 with isolated mTBI)	Mean age (years): 42.78 (all mTBI), 42.76 (isolated mTBI); male (%): 64.5 (all mTBI), 54.5 (isolated mTBI)	Patients at Turku University Hospital (Finland)	Not specified	Nf-L levels (<24 hours post-injury); CT results
Shahim et al. (2018) [25]	Prospective cohort	n = 192 (87 concussed, 74 contact-control, 19 non-athletic control, 12 gymnast control)	Median age (years): 26 (concussed), 28 (CC), 25 (NA) 19 (GC); male (%): not specified	Contact-sport athletes: Swedish Hockey League	Hockey	Nf-L levels (1, 12, 36, 144 hours post-injury, RTP); length of recovery (> or < 10 days)
Shahim et al. (2017) [24]	Prospective cohort	n = 42 (28 concussed, 14 non-athletic control)	Median age (years): 27 (concussed), 23.5 (NA); male (%): not specified	Contact sport athletes: Swedish Hockey League	Hockey	Nf-L levels (1, 12, 36, 144 hours post-injury); length of recovery (> or < 6 days)

The diagnostic criteria for mTBI varied among the selected articles, with some using guidelines established by organizations including the World Health Organization (WHO) or Department of Defense (DoD). Additionally, several studies assessed clinical symptoms following injury using various mTBI screening tools. Details can be found in Table 2.

**Table 2:** Diagnostic criteria and screening tools Diagnostic criteria and screening tools used to diagnose mTBI in each selected study. Abbreviations: LOC, loss of consciousness; PTA, post-traumatic amnesia; SCAT3, Sport Concussion Assessment Tool 3; WHO, World Health Organization; TBI, traumatic brain injury; GCS, Glasgow Coma Scale; DoD, Department of Defense; SAC, Standardized Assessment of Concussion Test.

Authors, year of publication	Diagnostic criteria	Symptom screening tool
Asken et al. (2020) [26]	Consensus criteria from the 4^th^ and 5^th^ International Conference on Concussion in Sport including one or more of the following: somatic (i.e. headache), cognitive (i.e. feeling like in a fog), and/or emotional symptoms (i.e. lability); physical signs (i.e. LOC/PTA/neurological deficit); balance impairment (i.e. gait unsteadiness); behavioral changes (i.e. irritability); cognitive impairment (i.e. slowed reaction times); sleep/wake disturbance (i.e. somnolence/drowsiness) [30,31]	SCAT3, King-Devick test
Clarke et al. (2021) [20]	WHO criteria: sustained a TBI, GCS score 13-15, no or <30 minutes of LOC, no or <24 hours of PTA [32]	Unspecified
Gill et al. (2018) [27]	Suspected TBI, GCS score 13-15	Unspecified
Giza et al. (2021) [28]	Consensus definition from the U.S. DoD evidence-based guidelines: potential concussive event, GCS score 13-15, <30 minutes LOC [33]	SCAT3, SAC
Hossain et al. (2019) [21]	Clinical diagnosis of TBI, GCS score 13-15	Unspecified
McCrea et al. (2020) [29]	Consensus definition from the U.S. DoD evidence-based guidelines: potential concussive event, GCS score 13-15, <30 minutes LOC [33]	SCAT3, SAC
Pattinson et al. (2020) [22]	Consensus definition from the U.S. DoD evidence-based guidelines: potential concussive event, GCS score 13-15, <30 minutes [33]; could also have observed or documented alterations in consciousness and/or mental state within 24 hours, <30 minutes of LOC, and/or <24 hours of PTA	Unspecified
Posti et al. (2019) [23]	Clinical diagnosis of TBI, GCS score 13-15	Unspecified
Shahim et al. (2018) [25]	Consensus criteria from the 4^th ^International Conference on Concussion in Sport including one or more of the following: somatic (i.e. headache), cognitive (i.e. feeling like in a fog), and/or emotional symptoms (i.e. lability); physical signs (i.e. LOC/PTA); behavioral changes (irritability); cognitive impairment (i.e. slowed reaction times); sleep disturbance (i.e. insomnia) [30]	Unspecified (although diagnostic criteria recommend the use of SCAT3 or SAC)
Shahim et al. (2017) [24]	Consensus criteria from the 4^th ^International Conference on Concussion in Sport including one or more of the following: somatic (i.e. headache), cognitive (i.e. feeling like in a fog), and/or emotional symptoms (i.e. lability); physical signs (i.e. LOC/PTA); behavioral changes (irritability); cognitive impairment (i.e. slowed reaction times); sleep disturbance (i.e. insomnia) [30]	Unspecified (although diagnostic criteria recommend the use of SCAT3 or SAC)

*Nf-L in the Acute Sett* *ing*

The relationship between Nf-L and mTBI diagnosis in the acute setting (within 24 hours of injury) served as the main outcome for this review. This acute temporal relationship was investigated by six studies, yielding contradicting results. Four of these studies used contact-sport athletes as participants, with three reporting significant acute elevations in Nf-L following mTBI. Asken et al. [26] measured Nf-L in a median of four hours post-injury and found significant Nf-L elevations in concussed athletes compared to contact-controls (median: 33.3 vs. 3.9 pg/ml, respectively, *p* = 0.01), with a sensitivity of 94.4% and area under the curve (AUC) of 0.9 (95% confidence interval (CI): 0.85-0.96) [26]. Shahim et al. (2018) [25] reported significantly higher Nf-L levels in concussed hockey players one-hour post-injury compared to non-concussed hockey players (*p* = 0.02), non-athletic controls (*p* = 0.03), and gymnast controls (*p* = 0.01) [25]. Similarly, Shahim et al. (2017) [24] found significantly elevated Nf-L levels in concussed hockey players at 12 hours post-injury compared to controls (*p* = 0.036) [24]. In contrast, McCrea et al. [29] did not find significant Nf-L elevations in concussed athletes within 24 hours of injury compared to both contact-controls (mean difference in natural log (ln) transformed values: 0.072 pg/ml, *p* = 0.628) and non-contact controls (mean difference in ln values: 0.039 pg/ml, *p* > 0.999). In fact, Nf-L was shown to have poor predictive value for a concussion at the acute time point with an AUC of 0.56 (95% CI: 0.48-0.63, *p* = 0.152) [29].

Giza et al. [28] examined this temporal relationship within the context of military personnel participating in combative training exercises. At the < six hours post-injury time point, no significant differences were observed between the concussed and contact-control cadets following post-hoc analysis. Such adjustments were necessary due to higher median Nf-L levels in the concussed group at baseline (mean difference in ln values: 0.309 pg/ml (95% CI: 0.105-0.513), *p* = 0.003) [28], presenting a possible confounding factor to the results of this study. Additionally, although involving the general population rather than athletes or military personnel specifically, Clarke et al. [20] also reported no significant difference in Nf-L levels within 24 hours of injury between emergency department patients with mTBI and controls (mean difference in log-transformed values: 0.06 (95% CI: -0.03-0.15), *p* = 0.19) [20].

Beyond the Acute Setting

Several previously mentioned studies also tracked Nf-L levels beyond 24 hours. Shahim et al. (2018) [25] found that while Nf-L levels declined temporarily 12 hours post-injury, levels steadily increased thereafter in concussed athletes compared to controls, peaking at 10 days post-injury (*p* = 0.0001) [25]. Shahim et al. (2017) [24] observed a significant peak in Nf-L levels at 144 hours post-injury (*p* = 0.045) in concussed athletes compared to controls, with levels returning to baseline by the return to play (RTP) [24]. While Clarke et al. [20] did not see a significant difference in Nf-L levels between mTBI and control patients at the 72-hour (mean difference in log-transformed values: 0.14 (95% CI: -0.09-0.37), *p* = 0.238) or 12-month (mean difference in log-transformed values: 0.01 (95% CI: -0.07-0.10), *p* = 0.735) time points, significant elevations were observed at two weeks and three months post-injury (mean difference in log-transformed values: 0.43 (95% CI: 0.26­-0.59) and 0.21 (95% CI: 0.14-0.28), respectively, *p* = 0.0001) [20]. Similarly, in regard to the acute time point, Giza et al. [28] found no significant differences in Nf-L levels between the concussed and contact-control cadets at the 24-48 hours post-injury, asymptomatic, and seven days following RTP time points [28]. McCrea et al. [29] did not find significant differences in Nf-L levels between concussed and contact or non-contact control athletes at the 24-48 hour (mean difference in ln values: 0.012 and -0.007 pg/ml, respectively, *p* > 0.999) or asymptomatic (mean difference in ln values: 0.023 and -0.026 pg/ml, respectively, *p* > 0.999) time points [29].

Immediate Complications of mTBI

A subset of patients experience loss of consciousness (LOC) and/or post-traumatic amnesia (PTA) immediately following an mTBI-causing head impact. McCrea et al. [29] found that Nf-L levels increased gradually over time in LOC-PTA athletes, reaching statistical significance upon symptom resolution. The LOC-PTA athletes had significantly higher levels at the asymptomatic time point compared to the no LOC-PTA athletes (mean difference in ln values: 0.290 pg/ml, *p* < 0.001) and contact-controls (mean difference in ln values: 0.248 pg/ml, *p* = 0.007). Such elevations in Nf-L persisted through the seven days post-RTP time point, with higher levels observed in the LOC-PTA athletes compared to the no LOC-PTA athletes and both the contact and non-contact controls (mean difference in ln values: 0.498, 0.481, and 0.448 pg/ml, respectively, *p *< 0.001) [29].

Patient Recovery

Several studies also revealed an association between Nf-L and recovery. Using Glasgow Outcome Scale-Extended (GOSE) patient scores six to 12 months post-injury, Hossain et al. [21] evaluated recovery status (complete recovery (GOSE = 8) vs. incomplete recovery (GOSE < 8); favorable outcome (GOSE = 5-8) vs. unfavorable outcome (GOSE = 1-4)). Nf-L levels within 24 hours of injury were found to be significantly higher in patients with incomplete recoveries (median: 17 vs. 11 pg/ml, AUC: 0.665 (95% CI: 0.561-0.768), *p* = 0.005) as well as in patients with unfavorable outcomes (median: 66 vs. 13 pg/ml, AUC: 0.826 (95% CI: 0.694-0.958), *p* < 0.001) [21]. Three additional studies evaluated recovery length. Shahim et al. (2018) [25] compared Nf-L levels between concussed athletes who returned to play in greater than (n = 49) or less than (n = 38) 10 days. Mean Nf-L levels were significantly greater across all time points for athletes in the RTP > 10 days group: 16.0 vs. 11.0 pg/ml (AUC: 0.82, OR: 8.8 (95%: CI 3.0-36.0), *p* = 0.006) at one hour; 14.0 vs. 11.0 pg/ml (AUC: 0.72, OR: 2.8 (95% CI: 1.3-7.3), *p* = 0.021) at 12 hours; 14.0 vs. 11.3 pg/ml (AUC: 0.73, OR: 3.0 (95% CI: 1.4-7.8), *p* = 0.011) at 36 hours; and 15.0 vs. 11.6 pg/ml (AUC: 0.73, OR: 3.3 (95% CI: 1.4-11.5), *p* = 0.025) at 144 hours. Nf-L had the greatest ability to distinguish athletes with longer recovery times at one-hour post-injury and was capable of identifying athletes who resigned from activity permanently due to unresolved symptoms at 144 hours post-injury (AUC: 0.89, *p* < 0.005) [25]. Shahim et al. (2017) [24] also used the above time points to compare concussed athletes who returned to play in greater than or less than six days. Significantly greater Nf-L levels were observed in athletes in the RTP > six days group across all four time points (*p* = 0.01-0.03), with minimal Nf-L fluctuation in those recovering in under six days. Nf-L was again found capable of distinguishing athletes with greater symptom duration, notably at one and 36 hours post-injury (AUC: 0.82 (95% CI: 0.6-1.0), *p* = 0.006 and AUC: 0.83 (95% CI: 0.6-1.0), *p* = 0.02, respectively) [24]. However, in contrast to two previous studies, Pattinson et al. [22] found Nf-L incapable of distinguishing athletes requiring greater than or less than 14 days recovery time following post-hoc analysis (mean difference within 21 hours of injury: 0.05 pg/ml (95% CI: -0.14-0.25), *p* = 0.59; mean difference 24-48 hours post-injury: 0.02 pg/ml (95% CI: -0.16-0.21), *p* = 0.81; mean difference once asymptomatic: 0.15 pg/ml (95% CI: -0.07-0.37), *p* = 0.17; and mean difference at seven days post-RTP: 0.20 pg/ml (95% CI: -0.03-0.44), *p* = 0.09) [22].

Neuroimaging Results

Three studies yielded conflicting results regarding the association between Nf-L and neuroimaging findings. Posti et al. [23] found that Nf-L was capable of distinguishing isolated mTBI patients based on CT results (AUC: 0.662 (95% CI: 0.512-0.812), *p* = 0.049), with a higher median Nf-L level in CT-positive patients (14.0 pg/ml) compared to CT-negative patients (8.23 pg/ml). The same was true for non-isolated mTBI patients (AUC: 0.676 (95% CI: 0.563-0.780), *p* = 0.004), with a median Nf-L level of 19.1 pg/ml in CT-positive patients and 13.0 pg/ml in CT-negative patients [23]. At first glance, Gill et al. [27] also observed a positive correlation between median Nf-L levels and positive neuroimaging results in mTBI patients: healthy controls: 4.87 pg/ml; MRI-CT-: 7.62 pg/ml; MRI+CT-: 17.94 pg/ml; and MRI+CT+: 23.20 pg/ml (*p* < 0.0001). However, upon stratifying patients based on CT results, no association existed between Nf-L and CT findings. In contrast, stratification based on MRI results revealed Nf-L as a significant predictor of positive MRI findings regardless of CT findings (AUC: 0.66, *p* = 0.02) and in patients with a known negative CT (AUC: 0.64, *p* = 0.015) [27]. This association was supported by Clarke et al. [20], which found a strong positive association between Nf-L levels and positive MRI results at two weeks and three months post-injury (*p* < 0.0001) [20].

Discussion

The potential use of Nf-L as a diagnostic biomarker for acute mTBI is a novel topic that up to this point has only served as the focus of a limited number of studies. To our knowledge, only six publications have specifically investigated this potential application within 24 hours of injury, and yet, these researchers have reached conflicting conclusions [20,24-26,28,29]. Of the four studies using athletes as subjects to evaluate Nf-L in the acute setting [24-26,29], three (75%) reported data supporting the efficacy of Nf-L in identifying mTBI within 24 hours of an injury [24-26]. However, neither of the two remaining studies using either military cadets or emergency department patients as subjects provided evidence supporting Nf-L use in the acute setting [20,28]. With only half of the current literature reporting significant acute elevations in Nf-L among mTBI patients regardless of the patient population, it remains difficult to determine the true diagnostic accuracy of Nf-L within 24 hours of injury. While Nf-L appears to have a relatively strong capability to distinguish mTBI-positive from mTBI-negative athletes acutely following injury, no efficacy is seen among military cadets or emergency department patients, limiting the ability to generalize these results to the general population. Perhaps there is a threshold for axonal membrane disturbance and Nf-L release that is not met in cases of uncomplicated mTBI, resulting in a minimal, insignificant change in serum Nf-L levels [34]. Higher Nf-L levels seen in patients with non-isolated mTBI may also indicate extracranial release of Nf-L from peripheral axons during injury [23]. False positive elevations in Nf-L may complicate the development of an accurate mTBI blood test in the future. Furthermore, the ideal time to measure acute Nf-L levels remains unclear, primarily due to the varying acute time points used by researchers, including one, four, six, 12, and 24 hours [20,24-26,28,29].

The optimal time at which to measure Nf-L following mTBI is further complicated by literature examining elevations beyond 24 hours post-injury [20,24,25,28,29]. Significant elevations in Nf-L observed by various studies at 144 hours, 10 days, two weeks, and three months post-injury make it difficult to identify the specific range of time Nf-L is elevated following injury [20,24,25]. However, such persistent elevations allude to the potential use of Nf-L to detect the previous mTBI, including those that went undiagnosed. Furthermore, Nf-L may have the potential to identify individuals with a history of multiple concussions or head traumas regardless of severity, as was seen in boxers who had higher Nf-L levels proportional to an increased number of repetitive subconcussive head impacts [24].

While the efficacy of Nf-L as an objective diagnostic tool in the acute setting is unclear, it may be better suited for predicting patient prognosis. The association between Nf-L and LOC or PTA may indicate a role of Nf-L in identifying mTBI of greater severity as neither is a universal complication of mTBI-causing injuries [29]. Combined with its ability to predict positive MRI results, elevations in Nf-L may signify the need for advanced imaging despite the traditional expectation of a lack of neuroimaging findings among mTBI patients [20,27]. Patients with significant Nf-L elevations may consequently benefit from a higher intensity of monitoring in a clinical setting with the goal of preventing progression to permanent neurocognitive dysfunction. Such long-term sequelae including chronic traumatic encephalopathy (CTE) remain a major concern among athletes and military personnel due to the increased exposure to repetitive traumatic head injuries. With high expectations placed on athletes and military personnel to return to activity as soon as possible following injury, Nf-L levels could potentially be considered by coaches and military leadership when determining recovery time allowances or the need for retirement or discharge due to likely unresolving symptoms [21,24,25].

Limitations

This systematic review has several limitations. As with all systematic reviews, there is a potential for bias due to the subjective nature of the literature search process. To limit such bias, at least two authors were involved in the selection of search keywords, outlining of inclusion and exclusion criteria, eligibility screening, quality assessment, data extraction, and the final selection of publications for analysis. Due to the limited number of publications on this topic as well as the small sample sizes and predominance of male subjects used within the selected studies, it was not possible to reach a definitive conclusion regarding the efficacy of Nf-L as an objective tool for the diagnosis of acute mTBI. Variation in the time points at which Nf-L was measured limits the ability to identify the ideal time at which to measure Nf-L post-injury. Furthermore, while the different patient populations included in the selected studies (contact-sport athletes, military personnel, emergency department patients, etc.) represent individuals disproportionately affected by mTBI, inconsistent results between subpopulations prevent the generalization of these results to the general population for the purposes of providing a recommendation regarding Nf-L use in the detection of mTBI in the acute setting or beyond.

Future perspectives for research

Given the limited number of completed studies, the potential for future research into the association between Nf-L and the diagnosis of acute mTBI is vast. However, future studies must address the weaknesses within the previous publications. Larger sample sizes with individuals from diverse backgrounds within athletics, the military, or the general population are required to ensure the results can be generalized to the entire population. A universal mTBI definition and diagnostic criteria as well as standardized acute time points of interest would allow for stronger comparisons between studies. Greater attention must also be given to the factors potentially contributing to the variation in baseline Nf-L levels between studies, such as age, sex, activity type, or previous injury. Finally, consideration of the type of assay used as well as the associated costs and availability within hospitals is essential to determining the viability and cost-effectiveness of the use of Nf-L as an acute mTBI biomarker.

## Conclusions

Due to the limited number of current publications investigating the potential role of Nf-L as a diagnostic biomarker for mTBI in the acute setting, the diagnostic efficacy and accuracy of Nf-L for this purpose cannot be determined. Nf-L shows promise as a prognostic tool for predicting complications associated with the initial injury, neuroimaging findings, and course of recovery in those diagnosed with mTBI. However, the ideal time at which the most significant changes in serum Nf-L should be observed and measured remains unclear. Additional studies are warranted to further investigate the use of Nf-L as the potential future of objective mTBI diagnosis in the clinical setting.

## References

[1] (2022). Centers for Disease Control and Prevention. Surveillance report of traumatic brain injury-related emergency department visits, hospitalizations, and deaths—United States. https://www.cdc.gov/traumaticbraininjury/pdf/TBI-Surveillance-Report-FINAL_508.pdf.

[2] Yengo-Kahn AM, Kelly PD, Liles DC (2020). The cost of a single concussion in American high school football: a retrospective cohort study. Concussion.

[3] Langlois JA, Rutland-Brown W, Wald MM (2006). The epidemiology and impact of traumatic brain injury: a brief overview. J Head Trauma Rehabil.

[4] Harmon KG, Drezner J, Gammons M (2013). American Medical Society for Sports Medicine position statement: concussion in sport. Clin J Sport Med.

[5] (2022). Centers for Disease Control and Prevention. TBI among service members and veterans. https://www.cdc.gov/traumaticbraininjury/military/index.html.

[6] Scorza KA, Cole W (2022). Current concepts in concussion: initial evaluation and management. Am Fam Physician.

[7] Giza CC, Hovda DA (2014). The new neurometabolic cascade of concussion. Neurosurgery.

[8] Bazarian JJ, Biberthaler P, Welch RD (2018). Serum GFAP and UCH-L1 for prediction of absence of intracranial injuries on head CT (ALERT-TBI): a multicentre observational study. Lancet Neurol.

[9] West TA, Marion DW (2014). Current recommendations for the diagnosis and treatment of concussion in sport: a comparison of three new guidelines. J Neurotrauma.

[10] (2022). Centers for Disease Control and Prevention. Concussion signs and symptoms. https://www.cdc.gov/headsup/basics/concussion_symptoms.html.

[11] McInnes K, Friesen CL, MacKenzie DE, Westwood DA, Boe SG (2017). Mild traumatic brain injury (mTBI) and chronic cognitive impairment: a scoping review. PLoS One.

[12] Arca KN, Starling AJ, Acierno MD, Demaerschalk BM, Marks L, O'Carroll CB (2020). Is King-Devick testing, compared with other sideline screening tests, superior for the assessment of sports-related concussion?: A critically appraised topic. Neurologist.

[13] Jeter CB, Hergenroeder GW, Hylin MJ, Redell JB, Moore AN, Dash PK (2013). Biomarkers for the diagnosis and prognosis of mild traumatic brain injury/concussion. J Neurotrauma.

[14] Papa L, Edwards D, Ramia M (2015). Exploring serum biomarkers for mild traumatic brain injury. Brain Neurotrauma: Molecular, Neuropsychological, and Rehabilitation Aspects.

[15] Kim HJ, Tsao JW, Stanfill AG (2018). The current state of biomarkers of mild traumatic brain injury. JCI Insight.

[16] Zetterberg H, Smith DH, Blennow K (2013). Biomarkers of mild traumatic brain injury in cerebrospinal fluid and blood. Nat Rev Neurol.

[17] Gaiottino J, Norgren N, Dobson R (2013). Increased neurofilament light chain blood levels in neurodegenerative neurological diseases. PLoS One.

[18] Page MJ, McKenzie JE, Bossuyt PM (2022). The PRISMA 2020 statement: an updated guideline for reporting systematic reviews. BMJ.

[19] (2022). Study Quality Assessment Tools. https://www.nhlbi.nih.gov/health-topics/study-quality-assessment-tools.

[20] Clarke GJ, Skandsen T, Zetterberg H (2021). One-year prospective study of plasma biomarkers from CNS in patients with mild traumatic brain injury. Front Neurol.

[21] Hossain I, Mohammadian M, Takala RS (2019). Early levels of glial fibrillary acidic protein and neurofilament light protein in predicting the outcome of mild traumatic brain injury. J Neurotrauma.

[22] Pattinson CL, Meier TB, Guedes VA (2020). Plasma biomarker concentrations associated with return to sport following sport-related concussion in collegiate athletes—a Concussion Assessment, Research, and Education (CARE) consortium study. JAMA Netw Open.

[23] Posti JP, Takala RS, Lagerstedt L (2019). Correlation of blood biomarkers and biomarker panels with traumatic findings on computed tomography after traumatic brain injury. J Neurotrauma.

[24] Shahim P, Zetterberg H, Tegner Y, Blennow K (2017). Serum neurofilament light as a biomarker for mild traumatic brain injury in contact sports. Neurology.

[25] Shahim P, Tegner Y, Marklund N, Blennow K, Zetterberg H (2018). Neurofilament light and tau as blood biomarkers for sports-related concussion. Neurology.

[26] Asken BM, Yang Z, Xu H (2020). Acute effects of sport-related concussion on serum glial fibrillary acidic protein, ubiquitin C-terminal hydrolase L1, total tau, and neurofilament light measured by a multiplex assay. J Neurotrauma.

[27] Gill J, Latour L, Diaz-Arrastia R (2018). Glial fibrillary acidic protein elevations relate to neuroimaging abnormalities after mild TBI. Neurology.

[28] Giza CC, McCrea M, Huber D (2021). Assessment of blood biomarker profile after acute concussion during combative training among US military cadets: a prospective study from the NCAA and US Department of Defense CARE consortium. JAMA Netw Open.

[29] McCrea M, Broglio SP, McAllister TW (2020). Association of blood biomarkers with acute sport-related concussion in collegiate athletes: findings from the NCAA and Department of Defense CARE consortium. JAMA Netw Open.

[30] McCrory P, Meeuwisse WH, Aubry M (2013). Consensus statement on concussion in sport: the 4th International Conference on Concussion in Sport held in Zurich, November 2012. Br J Sports Med.

[31] McCrory P, Meeuwisse W, Dvořák J (2017). Consensus statement on concussion in sport—the 5th International Conference on Concussion in Sport held in Berlin, October 2016. Br J Sports Med.

[32] Carroll LJ, Cassidy JD, Peloso PM (2004). Prognosis for mild traumatic brain injury: results of the WHO Collaborating Centre Task Force on Mild Traumatic Brain Injury. J Rehabil Med.

[33] Carney N, Ghajar J, Jagoda A (2014). Concussion guidelines step 1: systematic review of prevalent indicators. Neurosurgery.

[34] Wallace C, Zetterberg H, Blennow K, van Donkelaar P (2018). No change in plasma tau and serum neurofilament light concentrations in adolescent athletes following sport-related concussion. PLoS One.

